# Mechanism and clinical progression of solid tumors bone marrow metastasis

**DOI:** 10.3389/fphar.2024.1390361

**Published:** 2024-05-06

**Authors:** Ruohan Yang, Lin Jia, Jiuwei Cui

**Affiliations:** Cancer Center, The First Hospital of Jilin University, Changchun, China

**Keywords:** solid tumor, bone marrow metastases, clinical manifestations, prognosis, therapy regimens

## Abstract

The rich blood supply of the bone marrow provides favorable conditions for tumor cell proliferation and growth. In the disease’s early stages, circulating tumor cells can escape to the bone marrow and form imperceptible micro metastases. These tumor cells may be reactivated to regain the ability to grow aggressively and eventually develop into visible metastases. Symptomatic bone marrow metastases with abnormal hematopoiesis solid tumor metastases are rare and have poor prognoses. Treatment options are carefully chosen because of the suppression of bone marrow function. In this review, we summarized the mechanisms involved in developing bone marrow metastases from tumor cells and the clinical features, treatment options, and prognosis of patients with symptomatic bone marrow metastases from different solid tumors reported in the literature.

## 1 Introduction

Symptomatic bone marrow metastases (BMM) from solid tumors imply severe myelosuppression and a poorer prognosis. Bone marrow is a blood-rich soft connective tissue in the cancellous space of bone and the cavity of long bone marrow and is an essential source of hematopoietic cell production. Because of its unique environment, non-hematological solid tumor cells are less likely to invade the bone marrow. However, a tiny percentage of malignant tumor cells in extramedullary organs can metastasize via blood or lymphatic routes leading to symptomatic BMM (Wang et al., 2019). As normal bone marrow tissue is replaced by tumor tissue, patients usually present with suppression of hematopoietic functions such as anemia, thrombocytopenia, and abnormal coagulation. Some patients may also present with life-threatening disseminated intravascular coagulation (DIC) in gastric and colorectal cancers (Yoshioka et al., 1992; Nakashima et al., 2014; Hanamura et al., 2016; Seki and Wakaki, 2016; Zhai et al., 2022). Bone marrow aspiration biopsy(BMAB) reveals typical tumor cell infiltration and immunohistochemical staining helps to determine the origin of the tumor (Khan et al., 2019). Li et al. retrospectively studied 101 pathological specimens of patients with BMM, and the primary tumor sites were most common in the stomach (11 cases, 22%), lung (11 cases, 22%), and breast (9 cases, 18%) (Xiao et al., 2009). This is consistent with the findings of Hung et al. (2014).

In patients presenting with BMM, cytopenia frequently emerges as the principal clinical manifestation, constraining the dosage selection for chemotherapeutic agents and compromising the therapeutic efficacy (Kopp et al., 2011). Complicating the clinical landscape is the challenge of differentiating BMM from cytopenia induced by chemotherapy, a distinction that often eludes clinicians (Fumet et al., 2018). The bone marrow-blood barrier (MBB) further impedes the efficacy of certain chemotherapeutic drugs, particularly large molecules, which struggle to infiltrate the bone marrow milieu (Tavassoli, 2008). Owing to the covert progression of BMM, timely diagnosis and effective treatment are impeded, leading to a more dire prognosis for patients with solid tumor BMM as compared to those with metastases originating from other sites (Cardoso et al., 2020).

The published articles are mainly retrospective studies with small samples. Sakin et al. found that in 30 patients with BMM of breast cancer, the median overall survival (mOS) was 9 months (Sakin et al., 2020). And mOS of 31 weeks in 28 patients with small cell lung cancer (SCLC) with BMM (Zych et al., 1993). In a study of 39 cases of gastric cancer (GC) combined with BMM, the authors found that mOS was only 20–67 days (Kim et al., 2007).

Papac, in 1994, summarized the common tumor types of BMM and the application of new techniques for detecting tumors in the bone marrow (Papac, 1994). In recent years, as oncology treatment continues to evolve, the choice and application of drugs have become more diverse, and patients with BMM from solid tumors have gained survival benefits. In this review, we describe the factors involved in the bone marrow microenvironment that promote tumor metastasis and summarize the clinical features, treatment options, and prognosis of symptomatic BMM from different solid tumors.

## 2 Molecular mechanisms of BMM

**FIGURE 1:**
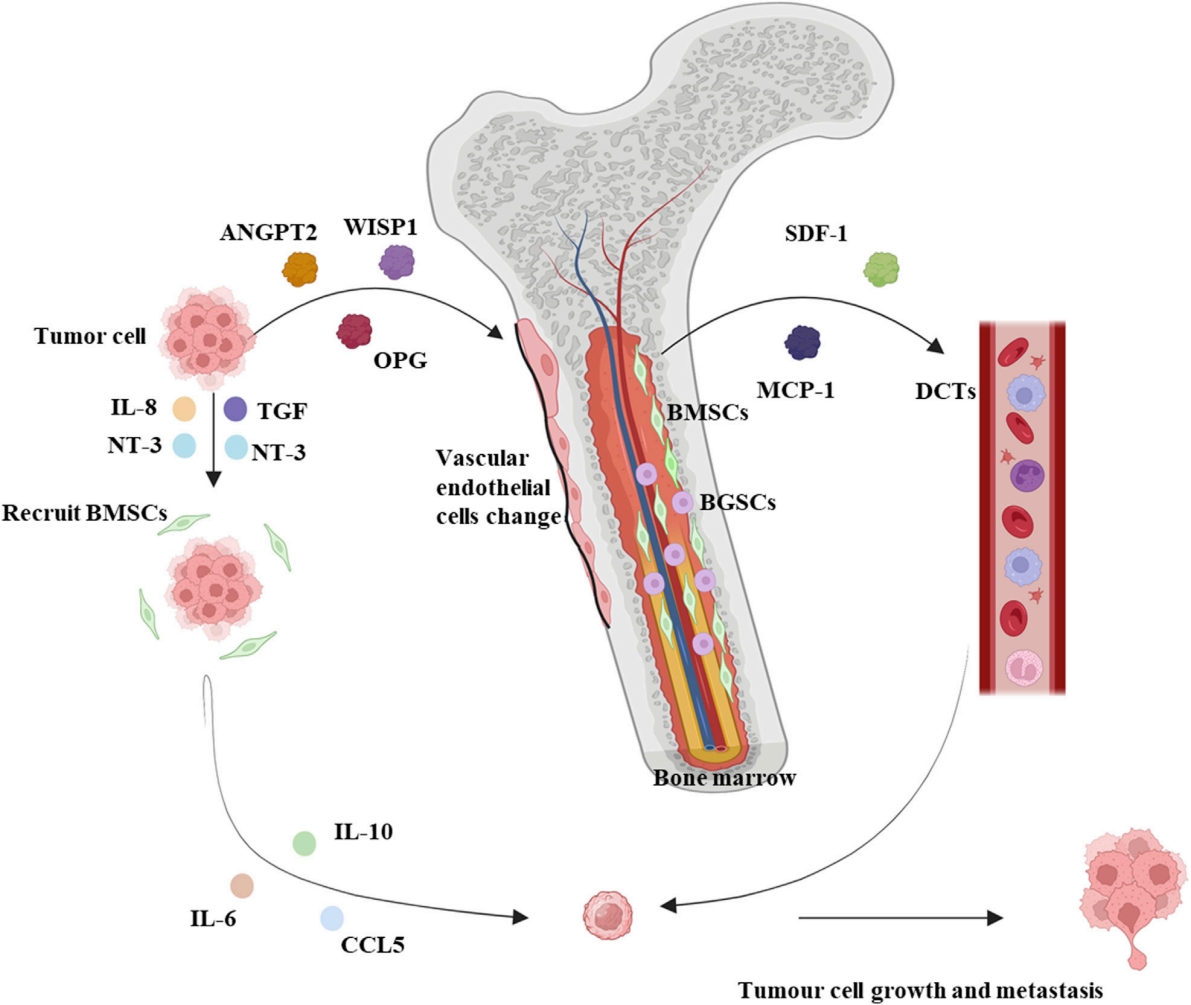
Summary mechanistic map between solid tumors and bone marrow microenvironment. Solid tumors lead to increased blood levels of proteins regulating bone tissue homeostasis such as ANGPT2, WISP1, and OPG, along with a reduction in the diameter volume and length of vascular endothelial cells, and MSCs are no longer distributed around the vascular endothelium, moving away from the blood vessels and near hematopoietic stem cells. Solid tumors secrete IL-8, TGF, and NT-3 to recruit BMSCs to the peritumoral area and release IL-6, IL-10, and CCL5 to promote tumor cell metastasis. Meanwhile, BMSCs promote the secretion of proteins such as SDF-1 and MCP-1, which stimulate circulating DCTs to promote tumor cell metastasis. BMSCs: Bone marrow-derived mesenchymal stem cells. BCSCs: Blood-generating stem cells. Interleukin: IL. DCTs: SDF-1: Stromal cell-derived factor-1. MCP-1: Monocyte chemoattractant protein-1. TGF: Tumor necrosis factor. CCL5: C-C chemokine ligand 5. NT3: Neurotrophin-3. Schematic in a created using BioRender (www.biorender.com).

BMM can be classified as micro metastases, symptomatic metastases, and bone marrow necrosis (BMN). Micro metastases are infiltrations and dormancy of circulating tumor cells (DCTs) in the bone marrow, which are not typical of patients’ symptoms and are easily overlooked by clinicians (Wang et al., 2019). The invasion of DCTs into blood vessels and the spread of blood circulation to organs throughout the body is the basic process of metastatic development, and DCTs are only “seeded” where “suitable soil” is available (Croucher et al., 2016). BM is characterized by having a large number of blood vessels, which allows tumor cells to enter the bone marrow cavity, BM microenvironment confers enhanced tumor metastasis capacity on tumor cells (Weilbaecher et al., 2011). Simultaneous recognition and interaction of adhesion molecules on tumor cells with bone marrow endothelial cells (ECs), stromal cells, and extracellular matrix. Adhesion to the BM endothelial intima also enhances tumor cell angiogenesis and bone resorption factor secretion, which favors the survival and growth of cancer cells (Weilbaecher et al., 2011). Bone metastases have been shown to occur more frequently in breast, prostate, and lung cancers (Coghlin and Murray, 2010). BM-derived hematopoietic stem cells expressing vascular endothelial growth factor receptor-1 (VEGFR-1) were demonstrated in a tumor-specific premetastatic niche and formed receptor clusters before the arrival of metastatic tumor cells in a mouse model. Blocking VEGFR-1 function specifically prevents the formation of premetastatic niches and tumor metastasis in BM (Kaplan et al., 2005; Kaplan et al., 2006a) (Figure 1).

Cytotoxic chemotherapy regimens will likely fail to eliminate such dormant, non-proliferating DTCs (Braun et al., 2003); this explains why some patients with early solid tumors develop distant metastases after several years (Ghajar et al., 2013). KAMBY et al. found microfiltration of tumor cells in the bone marrow of 87 (23%) of 320 patients with postoperative recurrent breast cancer (Kamby et al., 1987). Braun et al. also saw a poor prognosis for those with bone marrow micro metastases (Braun et al., 2005). Very rare symptomatic BMM resulting from early occult DCTs that spread hematogenous and invade highly vascularized bone marrow under certain conditions (Hung et al., 2014). When the expanding growth of proliferating tumor cells within the noncompliant space of the bone marrow cavity obstructs blood flow, leading to more severe ischemic BMN (Miyoshi et al., 2005). It is characterized by massive necrosis of the marrow and myeloid tissue of the hematopoietic bone marrow, forming an amorphous eosinophilic background, ill-defined necrotic cells, and preserved cortical bone (Maisel et al., 1988). BMN is very rare in solid tumors, and the prognosis for patients is poor (Wool and Deucher, 2015). The mechanisms of symptomatic bone marrow metastasis and bone marrow necrosis are unclear. Some studies have found that specific factors can promote bone marrow micro metastasis in solid tumor cells, so we summarized the molecular mechanisms.

### 2.1 Bone marrow-derived mesenchymal stem cells

Bone marrow-derived mesenchymal stem cells (BMSCs) influence the formation and development of tumor metastases. Mouse models of bone marrow metastasis confirm the involvement of BMSC in tumor invasion and metastasis (Kawai et al., 2018). BMSCs are a scarce cell type in the bone marrow, accounting for 0.01%–0.001% of all mononuclear cells (Pittenger et al., 1999). Studies have found that proteins regulating bone tissue homeostases such as Angiopoietin-2 (ANGPT2), WNT1-inducible-signalling pathway protein 1 (WISP1), and Osteoprotegerin (OPG) are increased in the blood of mice in a mouse model of breast cancer, and further studies have found that the morphology of the humeral blood vessels of mice with breast cancer is altered, as evidenced by a reduction in the diameter volume and length of vascular endothelial cells, and that MSCs are no longer distributed around the endothelium of the blood vessels, but are located away from the blood vessels and are close touched to the hematopoietic stem cells. This suggests that the endothelial ecological niche in which hematopoietic stem cells reside is remodeled. In addition, *in vitro* experiments have also shown that MSCs derived from mice with breast cancer can promote the expansion of hematopoietic stem cells and their differentiation to myeloid cells, affecting the myeloid system (Gerber-Ferder et al., 2023). Primary and metastatic tumor cells can release tumor necrosis factor (TGF), Interleukin(IL)-8, and Neurotrophin-3 (NT-3) to recruit BMSCs to the tumor site. BMSCs recruited to the tumor microenvironment differentiate into tumor-associated fibroblasts (TAF), which release IL-6, IL-10, C-C chemokine ligand 5 (CCL5), and extracellular matrix remodeling enzymes in the tumor microenvironment to affect tumor cell survival and angiogenesis (Bergfeld and DeClerck, 2010). In the bone marrow, however, BMSCs produce chemoattractant proteins such as stromal cell-derived factor-1(SDF-1) and monocyte chemoattractant protein-1 (MCP-1) that attract DCTs and promote tumor growth and drug resistance in the microenvironment (Bergfeld and DeClerck, 2010). Some reports suggest that BMSCs promote phagocytosis and vascularization of primary tumor development, thereby increasing the metastatic capacity of tumor cells (Kaplan et al., 2006b; Amé-Thomas et al., 2007) (Figure 1).

### 2.2 Bone marrow angiogenesis

The bone marrow is an extensively vascularized tissue, suggesting that blood vessels may play an essential role in the metastatic process of tumors (Kusumbe, 2016). It has been demonstrated that stable micro vessels in the bone marrow provide an ecological niche for dormant breast cancer cells. In a mouse model of breast cancer, dormant DCTs reside on the microvasculature of the bone marrow and sprout new vessels that stimulate the growth of breast tumor micro metastases (Ghajar et al., 2013). Similar results were found in the human study, where 19 of 42 patients (45%) with a bone marrow biopsy for breast cancer had bone marrow tumor cell infiltration. They found that patients with bone marrow micro metastases had significantly higher micro vessel density and had disease progression or recurrence at a substantially higher rate than patients with negative bone marrow puncture (Chavez-Macgregor et al., 2005).

Yip et al. (2021) found that in a mouse model of breast cancer, tumor cells preferentially enter a pre-existing epiphyseal domain rich in H-type blood vessels. Metastatic tumor growth can rapidly remodel the local microvascular system, establishing a microenvironment that promotes tumor growth (Yip et al., 2021). Similarly, it has been shown that endothelial cells and perivascular cells appear to promote the proliferation of tumor cells in BM in mouse models (Ghajar et al., 2013), These blood vessels release growth factors that promote tumor growth in mouse model BM (Ghajar, 2015).

### 2.3 Bone marrow chemokines

Chemokines secreted by different cells of the bone marrow play an essential role in forming the bone marrow ecotone system (Ahmadzadeh et al., 2015). Based on the position of the two conserved cysteine residues at the NH2 terminus, these low molecular weight peptides or proteins are classified into four groups, CXC, CC, C, and CX3C (Ahmadzadeh et al., 2015). In particular, the CXCL12-CXCR4 axis is vital in assisting the bone marrow in regulating tumor development (Shi et al., 2014). Chemokine CXCL12 is a highly conserved chemokine, and its receptor CXCR4 is a G protein-coupled receptor associated with intracellular heterotrimeric G proteins (Teicher and Fricker, 2010; Tanaka et al., 2012). CXCL12 and CXCR4 are involved in developing tumor progression and distant metastases and can lead to resistance to chemotherapy in solid tumors (Shi et al., 2014). One study found that CXCL12 expression in the bone marrow in a lung cancer model provided a displacement signal for CXCR4 tumor cells, increasing their invasion of the environment (Ahmadzadeh et al., 2015). In ovarian cancer, CXCR4 and CCR9 chemokine receptors cause resistance of tumor cells to apoptosis, promote their escape from the immune system, and increase angiogenesis (Singh et al., 2011). CXCR4, CCR2, and CX3CR1 are chemokines that play a prominent role in breast cancer metastasis to the bone marrow and in the proliferation of tumor cells (Müller et al., 2001; Lu and Kang, 2009; Jamieson-Gladney et al., 2011). The CXCR4 inhibitor AMD3100 has been shown to enhance the sensitivity of chemotherapy in a mouse model of multiple myeloma. At the same time, the CXCL12-CXCR4 axis can exert anti-tumor effects through inhibition of the CXCL12-CXCR4 axis (de Nigris et al., 2012). In addition to the CXCL12-CXCR4 axis, chemokines CCL12 and CCL22 enhance tumor cell formation in the bone marrow microenvironment and are involved in tumor transformation, growth, and metastasis (Kulbe et al., 2004).

### 2.4 Other factors in the bone marrow microenvironment

In addition, regulatory T cells (Tregs), closely associated with tumor development, are also widely stored in the bone marrow. It has been shown that Tregs infiltration into tumors is a poor prognostic marker and that depletion of Tregs in a 4T1 mouse model inhibits the development of lung metastases (Hong et al., 2010).

Bone marrow adipocytes (BMAs) are abundant in the bone marrow microenvironment and account for approximately 70% of the adult bone marrow volume (Luo et al., 2018). It has been suggested that BMAs may act as an energy source during tumor progression (Luo et al., 2018). BMAs secrete adipocytokines such as leptin, adiponectin, IL-1β, IL-6, VCAM-1, TNF-α, and VEGF to promote tumor cell metastasis (Shin and Koo, 2020).

## 3 Clinical features and diagnosis of patients with symptomatic BMM from solid tumors

**FIGURE 2:**
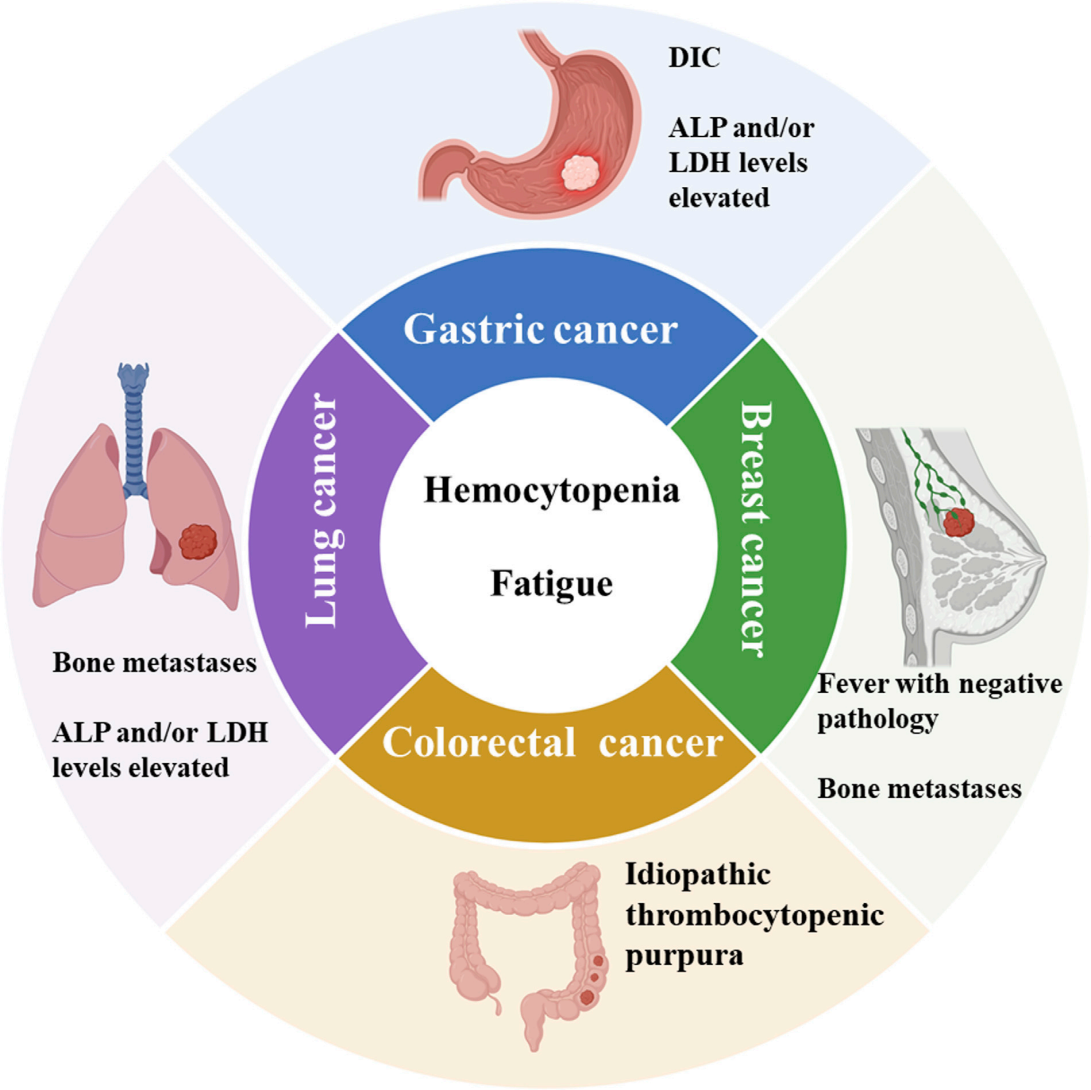
Summary of clinical features of bone marrow metastases from common solid tumors. ALP: Alkaline phosphatase. LDH: Lactate dehydrogenase. DIC: Disseminated intravascular coagulation. Schematic in a created using BioRender (www.biorender.com).

Li et al. found 101 cases (1.0%) of solid tumor metastases in a review of 10,112 bone marrow samples, with lung, gastric, breast, and prostate cancers being the most common. In addition to the typical peripheral blood changes described previously, this study found patients with non-specific clinical symptoms such as skeletal pain (24.75%) and unexplained fever (4.95%) (Xiao et al., 2009). The number of patients limits published studies, and clinical characteristics still need to be systematically summarized. We searched the published literature for clinical characteristics of patients with bone marrow metastases from different solid tumors such as gastric, lung, and breast cancer and summarized them (Figure 2).

### 3.1 Gastric cancer

Gastric cancer (GC) is the fifth most common cancer and the fourth leading cause of cancer-related death worldwide (Qiu et al., 2021; Sung et al., 2021). GC combination with BMM significantly shortens the survival (Kim et al., 2007). There is a lack of prospective studies in this area, with small retrospective samples and case reports predominating. In addition to typical peripheral blood changes such as anemia and thrombocytopenia, patients with GC are more likely to present clinically with DIC, and BMAB reveals tumor cells with markedly heterogeneous staining (Kim et al., 2007; Iguchi, 2015; Zhai et al., 2022). Kim et al. retrospectively studied 39 cases of BMM of GC. The clinical features included mostly young male patients with elevated serum alkaline phosphatase (ALP) and/or lactate dehydrogenase (LDH) levels in laboratory tests and pathological types of hypo-f fractionated adenocarcinoma or indolent cell carcinoma (Kim et al., 2007). This is consistent with the studies reported (Kwon et al., 2011; Iguchi, 2015; Zhai et al., 2022) (Table 1). It has been found that bone metastases tend to occur in the hematopoietic bone; therefore, bone marrow involvement is considered a prerequisite for bone metastases (Papac, 1994). Our literature review revealed that BMM of GC is most often associated with extensive bone metastases. Of the 39 patients with BMM, 69.23% had multiple bone metastases (Kim et al., 2007). In another study, 57.7% of patients had a combination of bone metastases (Kwon et al., 2011). Significant expression of RANKL, a primary regulator of osteoclast differentiation and activation, plays an essential role in the bone marrow dissemination of GC (Kusumoto et al., 2006).

**TABLE 1:** Summary of treatment modalities and prognosis of different solid tumors.

Treatment	Tumor type	Pathological type	Molecular type	Use of medications	OS/mOS	Results
Chemotherapy	Breast cancer	Invasive ductal carcinoma Invasive lobular carcinoma	Triple-negative HR positive, HER-2 negative Her-2 overexpression	Adriamycin, doxorubicin, cyclophosphamide	6–38 months	Chemotherapy significantly prolongs survival in breast cancer patients with bone marrow metastases. Among them, paclitaxel treatment achieved the best survival rate Freyer et al. (2000), Kopp et al. (2011), Sakin et al. (2020)
	Gastric cancer	Poorly differentiated/signet ring cell	NA	FOLFOX, FOLFIRI	67–137 days	Gastric cancer patients with bone marrow metastases should receive more tailored therapies for risk factors to enhance survival. Kim et al. (2007), Ubukata et al. (2011)
		Well/moderately differentiated				
	Gastric cancer	Hypo fractionated adenocarcinoma	NA	Tegafur	8.1 ± 2.7 months	Due to the low incidence of BMM in gastric cancer, it has not received sufficient attention, and the prognosis has not improved much Iguchi (2015)
		Indolent cell carcinoma, Tubular adenocarcinoma				
	Colorectal cancer	Moderately to poorly differentiated adenocarcinoma	NA	FOLFOX, FOLFIRI, Bevacizumab, 5-fluorouracil, Adriamycin	15 days-10 months	Clinical bone marrow involvement limits the clinician’s ability to tailor chemotherapy regimens. Early diagnosis is critical in future treatment and therapeutic decisions Ozkan et al. (2007), Arslan et al. (2015), Assi et al. (2016), Hanamura et al. (2016)
	Lung Cancer	Small cell lung cancer	NA	NA	31 week	The presence of BMM is associated with a shorter time to progression and survival Zych et al. (1993)
	Rhabdomyosarcoma	Embryonal, Alveolar	NA	NA	18–27 months	RMS with BMM has a low survival rate and new therapies are needed to alleviate the disease Aida et al. (2015), Bailey and Wexler (2020), Huang et al. (2021)
Endocrine therapy	Breast cancer	Invasive lobular carcinoma	HR positive, HER-2 negative	Palbociclib + Letrozole + ovarian suppression	26 months	A combination of endocrine therapy and CDK4/6 inhibitor may have more extended clinical benefit than chemotherapy, and a combination therapy of ET and CDK4/6 inhibitor is less toxic and leads to a better quality of life than chemotherapy Garufi et al. (2021)
	Breast cancer	Invasive lobular carcinoma	HR positive, HER-2 negative	Aromatase inhibitor	7 months	After hormonal treatment with the aromatase inhibitor, the patient’s condition improved Rahmat and Ikhwan (2018)
Target therapy	Breast cancer	Invasive ductal carcinoma	HER-2 overexpression	Trastuzumab	11 months	Trastuzumab may be a beneficial treatment option for patients with Her-2-positive bone marrow metastases Xu et al. (2014)
	Lung Cancer	lung adenocarcinoma	NA	Anti- EGFR therapy	422 days	Patients with BMM from lung adenocarcinoma have a short survival period, which can be prolonged by targeted drugs Wang et al. (2019)

NA, not applicable; HR, Hormone Receptor; FOLFOX, paclitaxel; 5-fluorouracil, calcium folinic acid, and oxaliplatin FOLFIRI:5-fluorouracil, calcium folinic acid, irinotecan. EGFR, Epidermal growth factor receptor. DIC, disseminated intravascular coagulation. mOS, median Overall survival.

### 3.2 Breast cancer

Studies have shown that symptomatic BMM is rare in metastatic breast cancer (Kopp et al., 2011). We screened the published literature and summarized the clinical characteristics of patients with BMM (Table 1). Kopp et al. retrospectively studied 22 patients with BMM of breast cancer, 50% (11/22) were molecularly typed as hormone receptor (HR) positive and human epidermal growth factor receptor 2 (HER-2) negative (Kopp et al., 2011), Sakin et al. found similar results (21/30, 70%) (Sakin et al., 2020). Invasive ductal carcinoma is the primary pathological type constituting BMM, followed by invasive lobular carcinoma (Kopp et al., 2011; Sakin et al., 2020), Documented histological grading of 2–3 (Bjelic-Radisic et al., 2006; Rahmat and Ikhwan, 2018). Patients mostly present with clinical weakness (Ardavanis et al., 2008; Akagi et al., 2021). Our previous study found nearly 76% of patients had a pathogenically negative fever. We also found a combination of bone metastases in all patients, with spinal metastases being the most common (78.78%) (Yang et al., 2022a). This may be due to higher levels of the chemokine CXC3L1/CXC3R1 in the spine than in other bones, which can promote adhesion and migration of breast cancer cells (Meng et al., 2022).

### 3.3 Colorectal cancer

Colorectal cancer is the third leading cause of cancer-related death, with common distant metastases usually occurring in the liver and lungs, and symptomatic BMM is extremely rare in colorectal cancer (Assi et al., 2016; Alghandour et al., 2020). Similar to BMM from other malignancies, the common signs and symptoms have been shown to include malaise, fever, and complete blood cytopenia (Assi et al., 2016). Occasionally life-threatening DIC and idiopathic thrombocytopenic purpura (TTP) (Lee et al., 2004; Hanamura et al., 2016). The published articles are case reports. The patient was an elderly male with a predominantly low to moderately differentiated adenocarcinoma of the rectum and sigmoid colon as the main pathological feature (Arslan et al., 2015; Assi et al., 2016; Alghandour et al., 2020). Only OZKAN et al. reported one patient with the pathology of a highly differentiated neuroendocrine tumor of the rectum combined with BMM (Table 1) (Ozkan et al., 2007).

### 3.4 Lung cancer

In recent years, radiotherapy, chemotherapy, and molecular targeted therapy have become essential treatment strategies for patients with advanced lung cancer (Jones and Baldwin, 2018). However, the prognosis for patients with BMM from lung adenocarcinoma remains unsatisfactory (Wang et al., 2019). Wang et al. retrospectively studied 12 patients with lung adenocarcinoma BMM and found that the patient population was predominantly middle-aged and older men. For the more malignant small cell lung cancer (SCLC) BMM, the patient population was also middle-aged and elderly males (21 cases, 75%) (Zych et al., 1993). The patients’ hematology showed decreased blood cells and ALP and LDH levels. They also found that most patients had a combination of bone metastases (Zych et al., 1993; Wang et al., 2019).

### 3.5 Other solid tumors

Apart from the solid tumors mentioned above, other solid tumors of BMM are less frequently reported. The published articles are retrospective studies of small samples, and we summarized their clinical features in the following sections.

#### 3.5.1 Prostate cancer

Prostate cancer is one of the most common malignancies in men worldwide and the leading cause of death in men worldwide, and metastatic prostate cancer implies a poor prognosis (Table 1) (Morash et al., 2015). There are few reports related to BMM in prostate cancer clinical features. Shahait et al. retrospectively studied 189 patients with prostate cancer, of whom 11 (6%) had a diagnosis of BMM (Shahait et al., 2022). BMAB reveals many scattered or clumped metastatic cancer cells with clinical features such as anemia, elevated ALP, and poor ECOG fitness status (Shahait et al., 2022).

#### 3.5.2 Rhabdomyosarcoma

Rhabdomyosarcoma (RMS) is the most common soft tissue sarcoma in adolescence and childhood (Bailey and Wexler, 2020). Bone marrow is a common site of distant metastases in RMS, with an incidence of 6% (Weiss et al., 2013). Some studies have confirmed that alveolar RMS is the most common type of pathology, with no significant specificity for age or sex (Table 1) (Bailey and Wexler, 2020; Huang et al., 2021).

For BMM in hepatocellular carcinoma, nasopharyngeal carcinoma, glioma, and renal carcinoma, the number of published articles is small, and they are all case reports, so we do not summarize their clinical features in this article.

## 4 Treatment options and prognosis

The prognosis of patients with advanced solid tumors has improved with advances in treatment options. However, the prognosis for patients with symptomatic BMM remains poor compared to metastases from other sites. Chemotherapy remains an important treatment option for BMM in the remaining solid tumors, except breast and lung adenocarcinoma. We summarized the treatment options and prognosis of patients with documented BMM from gastric, lung, breast, and colorectal cancers.

### 4.1 Chemotherapy

#### 4.1.1 Gastric cancer

Limited data on BMM treatment options for GC and poor patient prognosis (Iguchi, 2015). We summarized the reported treatment regimens and prognosis (Table 1). Dittus et al. reported one male BMM patient treated with epirubicin, oxaliplatin, and capecitabine (EOX) chemotherapy. The patient’s prognosis and the associated adverse events were not recorded at (Dittus et al., 2014). In a study of five patients who received a combination of platinum, 5-fluorouracil, and docetaxel chemotherapy, overall survival (OS) was only 20–53 days (Ekinci et al., 2014).

Studies have shown a survival benefit of chemotherapy in patients with BMM of GC. And mOS was 67 days in patients treated with paclitaxel, 5-fluorouracil, calcium folinic acid, and oxaliplatin (FOLFOX) and 5-fluorouracil, calcium folinic acid, irinotecan (FOLFIRI) chemotherapy with no treatment-related adverse events recorded by Kim et al. (2007). Kwon et al. found an mOS of 121 days in 16 patients receiving platinum, paclitaxel, irinotecan, and 5-fluorouracil chemotherapy, respectively. Patients had higher leukocyte, neutrophil, and platelet levels after cytotoxic treatment, improved bone marrow function, and no treatment-related deaths (Kwon et al., 2011). A 2015 study in Japan summarized 14 cases treated with Tegafur with OS for up to 8.1 months and well-tolerated by patients (Iguchi, 2015).

BMM of GC combined with DIC means a worse prognosis. An 80-year-old man diagnosed with BMM combined with DIC and thrombotic microangiopathy did not receive antineoplastic treatment and died on the third day after admission to hospital (Seki and Wakaki, 2016). Zhai et al. examined 36 patients with concurrent BMM and DIC receiving chemotherapy based on 5-fluorouracil, paclitaxel, and platinum drugs. They found that survival time after chemotherapy was strongly correlated with remission of DIC. mOS was 7.2 months in the DIC remission group and only 0.93 months in the no remission group (Zhai et al., 2022).

Our literature review found that Tegafur chemotherapy may be the best option for BMM in GC and may provide a survival benefit to patients. In the case of frequent life-threatening DIC, the effectiveness of chemotherapy is a critical factor in the survival time of patients. Future prospective studies are needed to compare the efficacy of different chemotherapeutic agents in BMM of GC.

#### 4.1.2 Small-cell lung cancer

Small cell lung cancer (SCLC) is highly malignant, and approximately 2/3 of SCLC patients have distant metastases to the brain, liver, adrenal glands, bone, and bone marrow at the time of initial diagnosis, with studies showing that the frequency of bone marrow involvement in SCLC ranges from 32% to 46% (Ihde et al., 1979). Zych et al. studied 28 patients with symptomatic BMM from SCLC treated with cyclophosphamide, Adriamycin, methotrexate, and etoposide. 60.7% of patients were assessed to be in complete or partial remission after chemotherapy, with an mOS of 31 weeks and no treatment-related adverse events recorded (Zych et al., 1993). In a study of 14 patients receiving cyclophosphamide chemotherapy, an mOS of 8 months was found. However, BMM patients required more red blood cell infusion, and up to 29% developed severe infections (sepsis and pneumonia) (Ihde et al., 1979). Asai et al. (2013) reported one case treated with small incremental doses of Adriamycin in which the patient remained in complete remission after a 6-month follow-up period. Still, unfortunately, the authors did not record data on his overall survival and adverse effects. Patients with SCLC combined with BMM have a poor prognosis, and small studies have not identified safe and effective treatment options for patients with SCLC combined with BMM.

#### 4.1.3 Breast cancer

Symptomatic BMM from breast cancer is rapidly progressive and has a poor prognosis (Cardoso et al., 2020). The 5th ESO-ESMO International Consensus Guidelines for Advanced Breast Cancer recommend chemotherapy for rapid and effective symptomatic relief regardless of the patient’s receptor expression (Cardoso et al., 2020). However, there are differences in treatment tolerability and prognosis with different drugs, which we have summarized.

Combining anthracyclines and anti-microtubule drugs is one of the most effective therapies for treating metastatic breast cancer (OBrien et al., 2004). It is also widely used in BMM. In a retrospective study of 22 patients, the best response rate was found in the adriamycin combined with the doxorubicin treatment group, as evidenced by increased white blood cells, platelets, and hemoglobin and an overall mOS of 11 months (Kopp et al., 2011). However, five patients developed febrile neutropenia, and four developed bleeding-related adverse events (three grade 3 and one grade 4) during treatment (Kopp et al., 2011). Another study found that one patient discontinued due to a severe adverse neurotoxic event during treatment with Adriamycin and cyclophosphamide in combination with docetaxel (Akagi et al., 2021). Pahouja et al. reported that a patient with BMM treated with adriamycin monotherapy survived 44 months. However, a granulocyte deficiency fever occurred in the second treatment cycle, reducing drug dosage (Pahouja et al., 2015). Sakin et al. found that of 30 patients with BMM of breast cancer, 18 treated with paclitaxel achieved the best survival with an mOS of 9.0 months and no treatment-related adverse events (Sakin et al., 2020).

In addition to the cytotoxic chemotherapeutic agents mentioned above, Ardavanis et al. reported on five patients with BMM treated with low-dose capecitabine oral chemotherapy, two patients with OS over 22 months and well-tolerated drug with no serious adverse events (Ardavanis et al., 2008). A 62-year-old female patient diagnosed with BMM due to thrombocytopenia was subsequently treated with docetaxel, Adriamycin, capecitabine, CMF, vincristine, gemcitabine, and carboplatin, respectively, and had OS of 57 months with increased platelet levels after treatment onset. However, multiple recurrent grade 3 or four neutropenia and leukopenia adverse events occurred during treatment (Bjelic-Radisic et al., 2006).

Cytotoxic chemotherapy delays the progression of BMM disease, and an increase in blood cell count is the main indication that the disease is under control. However, the hematological toxicity and neurotoxicity caused by chemotherapy cannot be ignored.

#### 4.1.4 Colorectal cancer

Chemotherapy is also the treatment of choice for BMM patients with colorectal cancer (Assi et al., 2016). The published articles were small sample studies with variable drug choices. Assi et al. reported on three patients with colon cancer combined with BMM. One was treated with 12 cycles of FOLFOX and achieved complete clinical remission. While the other two had an OS of 4 and 6 months, respectively, no adverse events from the drug were recorded (Assi et al., 2016). ÖZKAN et al. reported rectal cancer combined with BMM treated with systemic chemotherapy using a modified FOLFOX(mFOLFOX) regimen, in which the patient’s hematocrit improved after three treatment cycles. Bevacizumab combination therapy was added in cycle five. Still, the patient experienced significant fatigue and decreased ability to perform daily activities due to chemotherapy and died at 4 months of diagnosis (Nakashima et al., 2014). HANAMURA et al. reported a patient with sigmoid BMM treated with mFOLFOX, capecitabine in combination with oxaliplatin, irinotecan, and panitumumab, respectively, with survival of up to 10 months (Hanamura et al., 2016).

### 4.2 Molecular targeted therapy

In recent years, targeted drugs for different targets of malignant tumors have been introduced, significantly prolonging survival and improving quality of life (Lee et al., 2018a). For patients with HER-2 overexpression, molecularly targeted anti-HER-2 therapy substantially prolongs survival (Ekinci et al., 2014). Of the 12 patients with HER-2 overexpressing lung adenocarcinoma, they were treated with platinum-based chemotherapy, chemotherapy, and targeted drug tyrosine kinase inhibitor (TKI) therapy, TKI therapy alone or best supportive care in separate cases. TKI-targeted patients had a significantly better survival time than chemotherapy alone and palliative care (*p* = 0.031) (Wang et al., 2019). Wu et al. reported a case of a 62-year-old female patient with HER-2 overexpressed lung adenocarcinoma BMM combined with DIC who opted for molecularly targeted therapy with pyrrolizidine. After 2 months of treatment, the patient’s hematocrit symptoms improved (Wu et al., 2020). The authors did not count adverse events of the drug or the OS of patients. Molecularly targeted therapy also significantly prolonged survival in patients with HER-2 overexpressing metastatic breast cancer (Modi et al., 2020). XU et al. reported a case of a 41-year-old female with HR-negative, HER-2-positive breast cancer BMM who was first treated with trastuzumab followed by paclitaxel-concurrent chemotherapy; the patient had an OS of 19 months and no associated adverse events were recorded (Xu et al., 2014).

Sorafenib inhibits multiple targets of tumor cells (CRAF, BRAF, etc.) and tumor vessels (CRAF, VEGFR-2, etc.) and significantly treats advanced hepatocellular carcinoma (Cheng et al., 2020). In a case of symptomatic BMM in combination with HCC reported by Hong et al., the patient was treated with sorafenib molecular targeting. However, OS was 2.3 months due to the patient’s poor physical strength and discontinuation of treatment for diarrhea (Hong et al., 2016).

Targeted epidermal growth factor (EGFR) therapy also plays a prominent role in solid tumor BMM. Zhang et al. reported a case of a patient receiving gemcitabine, cisplatin, and cetuximab chemotherapy in combination with targeted therapy, sequential capecitabine, and sintilimab maintenance chemotherapy and immunotherapy. At publication, the patient had sustained complete remission of BMM (Zhang et al., 2022). A 45-year-old patient with rectal cancer BMM received cetuximab, FOLFIRI-targeted combination chemotherapy with partial disease remission after four cycles and OS of 8 months, with no treatment-related adverse events reported in either patient (Arslan et al., 2015).

### 4.3 Endocrine therapy/endocrine therapy combined with chemotherapy

Endocrine therapy (ET) is best for patients with HR positive, HER-2 negative breast cancer. Data are currently sparse in BMM due to its slow onset of action. One female BMM patient had an OS of 7 months after selecting a single aromatase inhibitor and tolerated treatment well (Rahmat and Ikhwan, 2018). Freyer et al. used tamoxifen in combination with or without gonadotropin-releasing hormone agonists or aromatase inhibitors in combination with weekly low-dose anthracycline chemotherapy in five patients with BMM breast cancer who had excellent disease control with OS of 12–38 months and no treatment-related adverse events (Freyer et al., 2000). It has been suggested that combining endocrine therapy and cell cycle protein-dependent kinase (CDK) 4/6 inhibitors may provide longer clinical benefits than chemotherapy in treating advanced breast cancer. Both the MONALEESA-3 (Slamon et al., 2021) and MONALEESA-7 (Lu et al., 2022) trials demonstrated that ribociclib combined with ET significantly prolonged disease-free progression survival and overall survival in patients with advanced HR positive, HER-2 negative breast cancer. Giovanna et al. reported a case of a woman receiving letrozole, leuprolide, and palbociclib for BMM of breast cancer who achieved a complete remission at 26 months (Garufi et al., 2021). Our previous study also found that of 33 patients with BMM breast cancer, 13 used ET combined with CDK4/6 inhibitors for mOS of 18.0 months, which was better than any previous study and had lower side effects (Yang et al., 2022a; Yang et al., 2022b). At the 2022 San Antonio Breast Cancer Symposium, the RIGHT Choice study found that receiving the CDK4/6 inhibitor ribociclib combined with ET in contrast to combination chemotherapy significantly prolonged PFS (24.0 vs. 12.3 months) in patients with visceral crisis, including those with symptomatic BMM, which was similar to our findings (Yang et al., 2022a; Yang et al., 2022b). This further supports the idea that CDK4/6 inhibitors in combination with ET could be the treatment of choice for breast cancer patients with HR positive, HER-2 negative.

## 5 Treatment modalities and prognosis of BMM in other solid tumors

Other solid tumors have a lower incidence of BMM, and fewer studies are available. The following summarized their treatment modalities and prognosis for different solid tumors.

### 5.1 Prostate cancer

Kaplan et al. (2012) reported a case of a patient with BMM of prostate cancer who received radiotherapy; however, tumor lysis syndrome occurred during radiotherapy with an OS of 11 days. In a study of 11 patients with prostate cancer combined with BMM, the authors found an mOS of 18.1 months after doxorubicin/abiraterone or systemic therapy, a significantly shorter survival time than the 42.2 months in patients without BMM (Shahait et al., 2022).

### 5.2 Rhabdomyosarcoma

Lee et al. retrospectively studied 51 pediatric patients with RMS and found that bone marrow involvement mOS was significantly shorter than in patients without BMM (17 vs. 61 months, *p* = 0.033). However, treatment modalities were not documented in the study (Lee et al., 2018). Bailey et al. found no significant improvement in prognosis despite patients receiving aggressive chemotherapy, with an mOS of approximately 18 months (Bailey and Wexler, 2020). And Huang et al., 2021 located in a single-center retrospective study that 13 patients with RMS combined with BMM received various treatment regimens, including chemotherapy, radiotherapy, and surgery, respectively, with an mOS of 27 months (Huang et al., 2021).

### 5.3 Glioblastoma multiforme

Less than 2% of patients with central nervous system (CNS) tumors are expected to develop extra-neurological metastases, with the bone marrow being an even rarer site for extra-neurological metastase (Didelot et al., 2006). Didelot et al. reported a glioblastoma multiforme (GBM) patient who presented with postoperative allogeneic cytopenia and subsequent BMM confirmed by BMAB. The patient was treated with one course of lomustine chemotherapy. However, the results were insignificant, with an OS of only 2 months (Didelot et al., 2006). Rajagopalan et al. reported a case of a 60-year-old man with BMM diagnosed after a BMAB for low back pain, thrombocytopenia, and hemoglobin reduction, who died after 1 month due to disease progression despite palliative radiotherapy (Rajagopalan et al., 2005). In the DEMASTER et al. study, patients did not receive anti-tumor therapy and died on day 5 and day 17 of the diagnosis of BMM, respectively (Kleinschmidt-Demasters, 1996).

### 5.4 Astrocytoma

LoRusso et al. first reported a patient with diffuse bone marrow involvement in astrocytoma who received concurrent intracranial radiotherapy and carmustine chemotherapy and died of sepsis 24 weeks after diagnosis (LoRusso et al., 1988). Hsu et al. found that patients treated with multiple lines of chemotherapy with etoposide, cyclophosphamide, cisplatin, carmustine, carboplatin, tamoxifen, and paclitaxel, respectively, had an OS of 21 months (Hsu et al., 1998).

### 5.5 Neuroblastoma

Hirano et al. reported a case of a patient presenting with left hip pain with hypothermia, initially diagnosed as septic osteomyelitis. However, subsequent negative pathogenic tests and aggressive anti-infective therapy did not significantly improve the fever symptoms. Further, BMAB confirmed a BMM from a neuroblastoma (Hirano et al., 2020). She was treated with chemotherapy, autologous peripheral blood hematopoietic stem cell transplantation, surgery, and radiation and went into remission. Overall survival time and adverse effects during treatment were not analyzed (Hirano et al., 2020).

### 5.6 Renal cell carcinoma

The common distant metastatic renal cell carcinoma organs are lungs, bones, and lymph nodes (Umer et al., 2018). BMM is very rare (Khan et al., 2019). Khan et al. identified a male patient with renal clear cell carcinoma in whom laboratory tests suggested that he did not have significant hematopoietic suppression. Cytopathy was found in the bone on computed tomography, and a bone marrow biopsy was performed, suggesting tumor cell infiltration. However, the authors did not report on the treatment and prognosis of patient (Khan et al., 2019).

## 6 Bone marrow necrosis

Hematological malignancies are the most common underlying disease of BMN, and caused by a solid tumor is very rare (Lee et al., 2004). Only two of the 101 smears of bone marrow metastases from solid tumors showed BMN (Xiao et al., 2009). Laboratory tests usually show suppression of bone marrow hematopoiesis (Wang et al., 2009). Common symptoms include fever, pancytopenia, and back pain (Janssens et al., 2000). This is similar to symptomatic BMM. In non-hematological malignancies, Lee et al. reported a case of a 67-year-old male with colorectal cancer BMN combined with thrombotic thrombocytopenic purpura, who received combination chemotherapy with oxaliplatin and 5-fluorouracil after diagnosis. After two treatment cycles, the patient’s hematological results improved. His hematology remains stable 4.5 months after diagnosis (Lee et al., 2004). However, a 37-year-old male with colon cancer BMN was treated with cetuximab in combination with oxaliplatin and fluorouracil. The patient’s disease went into remission in the first month after the start of treatment. Unfortunately, the patient died 3 months later due to disease progression (Wang et al., 2009).

## 7 Conclusion

In the early stages of malignancy, chemotherapy cannot wholly destroy the resting, dormant DCTs. At the same time, the bone marrow microenvironment with bone marrow-derived cells, microvasculature, and chemokines can promote the growth and metastasis of DCTs, resulting in symptomatic bone marrow metastases with allogeneic cytopenia in some patients. When the tumor cells in the bone marrow continue to proliferate and compress the microvasculature in the bone marrow, the result is impaired microcirculation and bone marrow necrosis. The suppression of bone marrow hematopoiesis and hemocytopenia characterizes this. DIC can also be life-threatening in some patients with gastric and colorectal cancers. Our literature review shows that most patients with solid tumors have BMM in combination with bone metastases, suggesting that bone marrow metastases may be a prerequisite for bone metastases. Symptomatic BMM and BMN have a poor prognosis. For patients with different types of tumors, cytotoxic chemotherapy can rapidly alleviate the symptoms of bone marrow infiltration. Still, its toxic side effects can significantly affect patients’ quality of life. We found that in patients with HER-2 overexpressed lung and breast cancers, chemotherapy combined with molecularly targeted therapy resulted in a survival benefit for patients with BMM, and in patients with HR positive, HER-2 negative breast cancers, CDK4/6 inhibitor with ET may be a better option for patients as its excellent great superiority due to its low toxicity and high efficiency. The bone marrow microenvironment can be disrupted by intervention to promote the metastatic drive of tumor cells. It is expected to prevent systemic metastasis in the later stage and control the tumor within a relatively easy treatment range. Future prospective studies with large samples are needed to explore the safety and efficacy of new agents in treating symptomatic BMM from different solid tumors.

## References

[B1] Ahmadzadeh A. Kast R. E. Ketabchi N. Shahrabi S. Shahjahani M. Jaseb K. (2015). Regulatory effect of chemokines in bone marrow niche. Cell Tissue Res. 361 (2), 401–410. 10.1007/s00441-015-2129-4 25715759

[B2] Aida Y. Ueki T. Kirihara T. Takeda W. Kurihara T. Sato K. (2015). Bone marrow metastasis of rhabdomyosarcoma mimicking acute leukemia: a case report and review of the literature. Intern Med. 54 (6), 643–650. 10.2169/internalmedicine.54.2473 25786457

[B3] Akagi H. Shimada A. Chin K. Domoto H. (2021). Successful stabilization of symptomatic bone marrow metastasis two times in a breast cancer patient. Anticancer Res. 41 (6), 3139–3144. 10.21873/anticanres.15099 34083308

[B4] Alghandour R. Saleh G. A. Shokeir F. A. Zuhdy M. (2020). Metastatic colorectal carcinoma initially diagnosed by bone marrow biopsy: a case report and literature review. J. Egypt Natl. Canc Inst. 32 (1), 30. 10.1186/s43046-020-00040-6 32676803 PMC13317082

[B5] Amé-Thomas P. Maby-El Hajjami H. Monvoisin C. Jean R. Monnier D. Caulet-Maugendre S. (2007). Human mesenchymal stem cells isolated from bone marrow and lymphoid organs support tumor B-cell growth: role of stromal cells in follicular lymphoma pathogenesis. Blood 109 (2), 693–702. 10.1182/blood-2006-05-020800 16985173

[B6] Ardavanis A. Kountourakis P. Orphanos G. Rigatos G. (2008). Low-dose capecitabine in breast cancer patients with symptomatic bone marrow infiltration: a case study. Anticancer Res. 28 (1b), 539–541. 18383899

[B7] Arslan C. Sen C. A. Ortac R. (2015). A case of rectal carcinoma with skin and bone marrow metastasis with concurrent extensive visceral involvement; unusual and dismal co-incidence. Expert Rev. Gastroenterol. Hepatol. 9 (6), 727–730. 10.1586/17474124.2015.1025053 25767005

[B8] Asai N. Ohkuni Y. Matsuda M. Narita M. Kaneko N. (2013). Incremental low doses of amrubicin for the treatment of bone marrow metastasis in small cell lung cancer. J. Bras. Pneumol. 39 (1), 108–110. 10.1590/s1806-37132013000100016 23503494 PMC4075794

[B9] Assi R. Mukherji D. Haydar A. Saroufim M. Temraz S. Shamseddine A. (2016). Metastatic colorectal cancer presenting with bone marrow metastasis: a case series and review of literature. J. Gastrointest. Oncol. 7 (2), 284–297. 10.3978/j.issn.2078-6891.2015.092 27034798 PMC4783735

[B10] Bailey K. A. Wexler L. H. (2020). Pediatric rhabdomyosarcoma with bone marrow metastasis. Pediatr. Blood Cancer 67 (5), e28219. 10.1002/pbc.28219 32100935 PMC7643423

[B11] Bergfeld S. A. DeClerck Y. A. (2010). Bone marrow-derived mesenchymal stem cells and the tumor microenvironment. Cancer Metastasis Rev. 29 (2), 249–261. 10.1007/s10555-010-9222-7 20411303

[B12] Bjelic-Radisic V. Stöger H. Winter R. Beham-Schmid C. Petru E. (2006). Long-term control of bone marrow carcinosis and severe thrombocytopenia with standard-dose chemotherapy in a breast cancer patient: a case report. Anticancer Res. 26 (2b), 1627–1630. 16619583

[B13] Braun S. Vogl F. D. Janni W. Marth C. Schlimok G. Pantel K. (2003). Evaluation of bone marrow in breast cancer patients: prediction of clinical outcome and response to therapy. Breast 12 (6), 397–404. 10.1016/s0960-9776(03)00143-7 14659112

[B14] Braun S. Vogl F. D. Naume B. Janni W. Osborne M. P. Coombes R. C. (2005). A pooled analysis of bone marrow micrometastasis in breast cancer. N. Engl. J. Med. 353 (8), 793–802. 10.1056/NEJMoa050434 16120859

[B15] Cardoso F. Paluch-Shimon S. Senkus E. Curigliano G. Aapro M. S. Andre F. (2020). 5th ESO-ESMO international consensus guidelines for advanced breast cancer (ABC 5). Ann. Oncol. 31 (12), 1623–1649. 10.1016/j.annonc.2020.09.010 32979513 PMC7510449

[B16] Chavez-Macgregor M. Aviles-Salas A. Green D. Fuentes-Alburo A. Gómez-Ruiz C. Aguayo A. (2005). Angiogenesis in the bone marrow of patients with breast cancer. Clin. Cancer Res. 11 (15), 5396–5400. 10.1158/1078-0432.Ccr-04-2420 16061853

[B17] Cheng Z. Wei-Qi J. Jin D. (2020). New insights on sorafenib resistance in liver cancer with correlation of individualized therapy. Biochim. Biophys. Acta Rev. Cancer 1874 (1), 188382. 10.1016/j.bbcan.2020.188382 32522600

[B18] Coghlin C. Murray G. I. (2010). Current and emerging concepts in tumour metastasis. J. Pathol. 222 (1), 1–15. 10.1002/path.2727 20681009

[B19] Croucher P. I. McDonald M. M. Martin T. J. (2016). Bone metastasis: the importance of the neighbourhood. Nat. Rev. Cancer 16 (6), 373–386. 10.1038/nrc.2016.44 27220481

[B20] de Nigris F. Schiano C. Infante T. Napoli C. (2012). CXCR4 inhibitors: tumor vasculature and therapeutic challenges. Recent Pat. Anticancer Drug Discov. 7 (3), 251–264. 10.2174/157489212801820039 22376154

[B21] Didelot A. Taillandier L. Grignon Y. Vespignani H. Beauchesne P. (2006). Concomitant bone marrow metastasis of a glioblastoma multiforme revealed at the diagnosis. Acta Neurochir. (Wien) 148 (9), 997–1000. 10.1007/s00701-006-0854-x 16932995

[B22] Dittus C. Mathew H. Malek A. Negroiu A. (2014). Bone marrow infiltration as the initial presentation of gastric signet ring cell adenocarcinoma. J. Gastrointest. Oncol. 5 (6), E113–E116. 10.3978/j.issn.2078-6891.2014.050 25436133 PMC4226827

[B23] Ekinci A. S. Bal O. Ozatli T. Turker I. Esbah O. Demirci A. (2014). Gastric carcinoma with bone marrow metastasis: a case series. J. Gastric Cancer 14 (1), 54–57. 10.5230/jgc.2014.14.1.54 24765538 PMC3996250

[B24] Freyer G. Ligneau B. Trillet-Lenoir V. V. (2000). Palliative hormone therapy, low-dose chemotherapy, and bisphosphonate in breast cancer patients with bone marrow involvement and pancytopenia: report of a pilot experience. Eur. J. Intern. Med. 11 (6), 329–333. 10.1016/s0953-6205(00)00121-7 11113657

[B25] Fumet J. D. Wickre M. Jacquot J. P. Bizollon M. H. Melis A. Vanoli A. (2018). Successfully treatment by eribulin in visceral crisis: a case of lymphangitic carcinomatosis from metastatic breast cancer. BMC Cancer 18 (1), 839. 10.1186/s12885-018-4725-7 30126360 PMC6102904

[B26] Garufi G. Carbognin L. Orlandi A. Palazzo A. Tortora G. Bria E. (2021). The therapeutic challenge of disseminated bone marrow metastasis from HR-positive HER2-negative breast cancer: case report and review of the literature. Front. Oncol. 11, 651723. 10.3389/fonc.2021.651723 34692469 PMC8529000

[B27] Gerber-Ferder Y. Cosgrove J. Duperray-Susini A. Missolo-Koussou Y. Dubois M. Stepaniuk K. (2023). Breast cancer remotely imposes a myeloid bias on haematopoietic stem cells by reprogramming the bone marrow niche. Nat. Cell Biol. 25 (12), 1736–1745. 10.1038/s41556-023-01291-w 38036749

[B28] Ghajar C. M. (2015). Metastasis prevention by targeting the dormant niche. Nat. Rev. Cancer 15 (4), 238–247. 10.1038/nrc3910 25801619 PMC4842412

[B29] Ghajar C. M. Peinado H. Mori H. Matei I. R. Evason K. J. Brazier H. (2013). The perivascular niche regulates breast tumour dormancy. Nat. Cell Biol. 15 (7), 807–817. 10.1038/ncb2767 23728425 PMC3826912

[B30] Hanamura F. Shibata Y. Shirakawa T. Kuwayama M. Oda H. Ariyama H. (2016). Favorable control of advanced colon adenocarcinoma with severe bone marrow metastasis: a case report. Mol. Clin. Oncol. 5 (5), 579–582. 10.3892/mco.2016.1029 27900088 PMC5103869

[B31] Hirano N. Goto H. Suenobu S. Ihara K. (2020). Bone marrow metastasis of neuroblastoma mimicking purulent osteomyelitis. Jpn. J. Clin. Oncol. 50 (10), 1227–1228. 10.1093/jjco/hyaa046 32310274

[B32] Hong H. Gu Y. Zhang H. Simon A. K. Chen X. Wu C. (2010). Depletion of CD4+CD25+ regulatory T cells enhances natural killer T cell-mediated anti-tumour immunity in a murine mammary breast cancer model. Clin. Exp. Immunol. 159 (1), 93–99. 10.1111/j.1365-2249.2009.04018.x 19817769 PMC2802699

[B33] Hong Y. M. Yoon K. T. Cho M. Kang D. H. Kim H. W. Choi C. W. (2016). Bone marrow metastasis presenting as bicytopenia originating from hepatocellular carcinoma. Clin. Mol. Hepatol. 22 (2), 267–271. 10.3350/cmh.2015.0017 27184470 PMC4946406

[B34] Hsu E. Keene D. Ventureyra E. Matzinger M. A. Jimenez C. Wang H. S. (1998). Bone marrow metastasis in astrocytic gliomata. J. Neurooncol 37 (3), 285–293. 10.1023/a:1005909127196 9524086

[B35] Huang C. Jian B. Su Y. Xu N. Yu T. He L. (2021). Clinical features and prognosis of paediatric rhabdomyosarcoma with bone marrow metastasis: a single Centre experiences in China. BMC Pediatr. 21 (1), 463. 10.1186/s12887-021-02904-9 34670517 PMC8529763

[B36] Hung Y. S. Chou W. C. Chen T. D. Chen T. C. Wang P. N. Chang H. (2014). Prognostic factors in adult patients with solid cancers and bone marrow metastases. Asian Pac J. Cancer Prev. 15 (1), 61–67. 10.7314/apjcp.2014.15.1.61 24528082

[B37] Iguchi H. (2015). Recent aspects for disseminated carcinomatosis of the bone marrow associated with gastric cancer: what has been done for the past, and what will be needed in future? World J. Gastroenterol. 21 (43), 12249–12260. 10.3748/wjg.v21.i43.12249 26604634 PMC4649110

[B38] Ihde D. C. Simms E. B. Matthews M. J. Cohen M. H. Bunn P. A. Minna J. D. (1979). Bone marrow metastases in small cell carcinoma of the lung: frequency, description, and influence on chemotherapeutic toxicity and prognosis. Blood 53 (4), 677–686. 10.1182/blood.v53.4.677.bloodjournal534677 218597

[B39] Jamieson-Gladney W. L. Zhang Y. Fong A. M. Meucci O. Fatatis A. (2011). The chemokine receptor CX₃CR1 is directly involved in the arrest of breast cancer cells to the skeleton. Breast Cancer Res. 13 (5), R91. 10.1186/bcr3016 21933397 PMC3262203

[B40] Janssens A. M. Offner F. C. Van Hove W. Z. (2000). Bone marrow necrosis. Cancer 88 (8), 1769–1780. 10.1002/(sici)1097-0142(20000415)88:8<1769::aid-cncr3>3.3.co;2-8 10760751

[B41] Jones G. S. Baldwin D. R. (2018). Recent advances in the management of lung cancer. Clin. Med. (Lond) 18 (Suppl. 2), s41–s46. 10.7861/clinmedicine.18-2-s41 29700092 PMC6334032

[B42] Kamby C. Guldhammer B. Vejborg I. Rossing N. Dirksen H. Daugaard S. (1987). The presence of tumor cells in bone marrow at the time of first recurrence of breast cancer. Cancer 60 (6), 1306–1312. 10.1002/1097-0142(19870915)60:6<1306::aid-cncr2820600624>3.0.co;2-x 3621113

[B43] Kaplan M. A. Kucukoner M. Alpagat G. Isikdogan A. (2012). Tumor lysis syndrome during radiotherapy for prostate cancer with bone and bone marrow metastases without visceral metastasis. Ann. Saudi Med. 32 (3), 306–308. 10.5144/0256-4947.2012.306-308 22588444 PMC6081029

[B44] Kaplan R. N. Psaila B. Lyden D. (2006b). Bone marrow cells in the 'pre-metastatic niche': within bone and beyond. Cancer metastasis Rev. 25 (4), 521–529. 10.1007/s10555-006-9036-9 17186383

[B45] Kaplan R. N. Rafii S. Lyden D. (2006a). Preparing the "soil": the premetastatic niche. Cancer Res. 66 (23), 11089–11093. 10.1158/0008-5472.Can-06-2407 17145848 PMC2952469

[B46] Kaplan R. N. Riba R. D. Zacharoulis S. Bramley A. H. Vincent L. Costa C. (2005). VEGFR1-positive haematopoietic bone marrow progenitors initiate the pre-metastatic niche. Nature 438 (7069), 820–827. 10.1038/nature04186 16341007 PMC2945882

[B47] Kawai H. Tsujigiwa H. Siar C. H. Nakano K. Takabatake K. Fujii M. (2018). Characterization and potential roles of bone marrow-derived stromal cells in cancer development and metastasis. Int. J. Med. Sci. 15 (12), 1406–1414. 10.7150/ijms.24370 30275769 PMC6158661

[B48] Khan S. Awan S. A. Jahangir S. Kamran S. Ahmad I. N. (2019). Bone marrow metastasis in clear cell renal cell carcinoma: a case study. Cureus 11 (3), e4181. 10.7759/cureus.4181 31106081 PMC6504026

[B49] Kim H. S. Yi S. Y. Jun H. J. Lee J. Park J. O. Park Y. S. (2007). Clinical outcome of gastric cancer patients with bone marrow metastases. Oncology 73 (3-4), 192–197. 10.1159/000127386 18418012

[B50] Kleinschmidt-Demasters B. K. (1996). Diffuse bone marrow metastases from glioblastoma multiforme: the role of dural invasion. Hum. Pathol. 27 (2), 197–201. 10.1016/s0046-8177(96)90376-7 8617464

[B51] Kopp H. G. Krauss K. Fehm T. Staebler A. Zahm J. Vogel W. (2011). Symptomatic bone marrow involvement in breast cancer--clinical presentation, treatment, and prognosis: a single institution review of 22 cases. Anticancer Res. 31 (11), 4025–4030. 22110237

[B52] Kulbe H. Levinson N. R. Balkwill F. Wilson J. L. (2004). The chemokine network in cancer--much more than directing cell movement. Int. J. Dev. Biol. 48 (5-6), 489–496. 10.1387/ijdb.041814hk 15349823

[B53] Kusumbe A. P. (2016). Vascular niches for disseminated tumour cells in bone. J. Bone Oncol. 5 (3), 112–116. 10.1016/j.jbo.2016.04.003 27761369 PMC5063228

[B54] Kusumoto H. Haraguchi M. Nozuka Y. Oda Y. Tsuneyoshi M. Iguchi H. (2006). Characteristic features of disseminated carcinomatosis of the bone marrow due to gastric cancer: the pathogenesis of bone destruction. Oncol. Rep. 16 (4), 735–740. 10.3892/or.16.4.735 16969487

[B55] Kwon J. Y. Yun J. Kim H. J. Kim K. H. Kim S. H. Lee S. C. (2011). Clinical outcome of gastric cancer patients with bone marrow metastases. Cancer Res. Treat. 43 (4), 244–249. 10.4143/crt.2011.43.4.244 22247710 PMC3253867

[B56] Lee D. H. Park C. J. Jang S. Cho Y. U. Seo J. J. Im H. J. (2018b). Clinical and cytogenetic profiles of rhabdomyosarcoma with bone marrow involvement in Korean children: a 15-year single-institution experience. Ann. Lab. Med. 38 (2), 132–138. 10.3343/alm.2018.38.2.132 29214757 PMC5736672

[B57] Lee J. L. Lee J. H. Kim M. K. Cho H. S. Bae Y. K. Cho K. H. (2004). A case of bone marrow necrosis with thrombotic thrombocytopenic purpura as a manifestation of occult colon cancer. Jpn. J. Clin. Oncol. 34 (8), 476–480. 10.1093/jjco/hyh082 15371467

[B58] Lee Y. T. Tan Y. J. Oon C. E. (2018a). Molecular targeted therapy: treating cancer with specificity. Eur. J. Pharmacol. 834, 188–196. 10.1016/j.ejphar.2018.07.034 30031797

[B59] LoRusso P. M. Tapazoglou E. Zarbo R. J. Cullis P. A. Austin D. Al-Sarraf M. (1988). Intracranial astrocytoma with diffuse bone marrow metastasis: a case report and review of the literature. J. Neurooncol 6 (1), 53–59. 10.1007/bf00163541 3294352

[B60] Lu X. Kang Y. (2009). Chemokine (C-C motif) ligand 2 engages CCR2+ stromal cells of monocytic origin to promote breast cancer metastasis to lung and bone. J. Biol. Chem. 284 (42), 29087–29096. 10.1074/jbc.M109.035899 19720836 PMC2781454

[B61] Lu Y. S. Im S. A. Colleoni M. Franke F. Bardia A. Cardoso F. (2022). Updated overall survival of ribociclib plus endocrine therapy versus endocrine therapy alone in pre- and perimenopausal patients with hr+/HER2- advanced breast cancer in MONALEESA-7: a phase III randomized clinical trial. Clin. cancer Res. official J. Am. Assoc. Cancer Res. 28 (5), 851–859. 10.1158/1078-0432.CCR-21-3032 PMC9377723 34965945

[B62] Luo G. He Y. Yu X. (2018). Bone marrow adipocyte: an intimate partner with tumor cells in bone metastasis. Front. Endocrinol. (Lausanne) 9, 339. 10.3389/fendo.2018.00339 30013512 PMC6036292

[B63] Maisel D. Lim J. Y. Pollock W. J. Yatani R. Liu P. I. (1988). Bone marrow necrosis: an entity often overlooked. Ann. Clin. Lab. Sci. 18 (2), 109–115. 3382156

[B64] Meng Q. Zhou L. Liang H. Hu A. Zhou H. Zhou J. (2022). Spine-specific downregulation of LAPTM5 expression promotes the progression and spinal metastasis of estrogen receptor-positive breast cancer by activating glutamine-dependent mTOR signaling. Int. J. Oncol. 60 (4), 47. 10.3892/ijo.2022.5337 35294039 PMC8923652

[B65] Miyoshi I. Daibata M. Ohtsuki Y. Taguchi H. (2005). Bone marrow necrosis. Br. J. Haematol. 130 (4), 467. 10.1111/j.1365-2141.2005.05532.x 16098058

[B66] Modi S. Saura C. Yamashita T. Park Y. H. Kim S. B. Tamura K. (2020). Trastuzumab deruxtecan in previously treated HER2-positive breast cancer. N. Engl. J. Med. 382 (7), 610–621. 10.1056/NEJMoa1914510 31825192 PMC7458671

[B67] Morash C. Tey R. Agbassi C. Klotz L. McGowan T. Srigley J. (2015). Active surveillance for the management of localized prostate cancer: guideline recommendations. Can. Urol. Assoc. J. 9 (5-6), 171–178. 10.5489/cuaj.2806 26225165 PMC4479637

[B68] Müller A. Homey B. Soto H. Ge N. Catron D. Buchanan M. E. (2001). Involvement of chemokine receptors in breast cancer metastasis. Nature 410 (6824), 50–56. 10.1038/35065016 11242036

[B69] Nakashima Y. Takeishi K. Guntani A. Tsujita E. Yoshinaga K. Matsuyama A. (2014). Rectal cancer with disseminated carcinomatosis of the bone marrow: report of a case. Int. Surg. 99 (5), 518–522. 10.9738/INTSURG-D-13-00130.1 25216414 PMC4253917

[B70] OBrien M. E. Wigler N. Inbar M. Rosso R. Grischke E. Santoro A. (2004). Reduced cardiotoxicity and comparable efficacy in a phase III trial of pegylated liposomal doxorubicin HCl (CAELYX/Doxil) versus conventional doxorubicin for first-line treatment of metastatic breast cancer. Ann. Oncol. 15 (3), 440–449. 10.1093/annonc/mdh097 14998846

[B71] Ozkan M. Er O. Karahan I. O. Deniz K. Coşkun R. Küçük C. (2007). Rectal carcinoid tumor with bone marrow and osteoblastic bone metastasis: a case report. Turk J. Gastroenterol. 18 (2), 111–114. 17602360

[B72] Pahouja G. Wesolowski R. Reinbolt R. Tozbikian G. Berger M. Mangini N. (2015). Stabilization of bone marrow infiltration by metastatic breast cancer with continuous doxorubicin. Cancer Treat. Commun. 3, 28–32. 10.1016/j.ctrc.2014.11.002 25914871 PMC4408922

[B73] Papac R. J. (1994). Bone marrow metastases. A review. Cancer 74 (9), 2403–2413. 10.1002/1097-0142(19941101)74:9<2403::Aid-cncr2820740904>3.0.Co;2-f 7922993

[B74] Pittenger M. F. Mackay A. M. Beck S. C. Jaiswal R. K. Douglas R. Mosca J. D. (1999). Multilineage potential of adult human mesenchymal stem cells. Science 284 (5411), 143–147. 10.1126/science.284.5411.143 10102814

[B75] Qiu H. Cao S. Xu R. (2021). Cancer incidence, mortality, and burden in China: a time-trend analysis and comparison with the United States and United Kingdom based on the global epidemiological data released in 2020. Cancer Commun. (Lond) 41 (10), 1037–1048. 10.1002/cac2.12197 34288593 PMC8504144

[B76] Rahmat C. Ikhwan R. (2018). Hormonal treatment for symptomatic bone marrow metastasis in breast cancer patients. Maedica (Bucur) 13 (3), 238–240. 10.26574/maedica.2018.13.3.238 30568745 PMC6290185

[B77] Rajagopalan V. El Kamar F. G. Thayaparan R. Grossbard M. L. (2005). Bone marrow metastases from glioblastoma multiforme--A case report and review of the literature. J. Neurooncol 72 (2), 157–161. 10.1007/s11060-004-3346-y 15925996

[B78] Sakin A. Sakalar T. Sahin S. Yasar N. Demir C. Geredeli C. (2020). Factors affecting survival and treatment efficacy in breast cancer patients with bone marrow metastasis. Breast J. 26 (4), 815–818. 10.1111/tbj.13647 31562662

[B79] Seki Y. Wakaki K. (2016). Pathological findings in a case of bone marrow carcinosis due to gastric cancer complicated by disseminated intravascular coagulation and thrombotic microangiopathy. Int. J. Hematol. 104 (4), 506–511. 10.1007/s12185-016-2051-x 27357318

[B80] Shahait M. Abu-Hijlih R. Salamat A. Abou Heidar N. Sharaf B. Abuhijla F. (2022). Bone marrow involvement in patients with metastatic castration sensitive prostate cancer. PLoS One 17 (7), e0270956. 10.1371/journal.pone.0270956 35862364 PMC9302741

[B81] Shi J. Wei Y. Xia J. Wang S. Wu J. Chen F. (2014). CXCL12-CXCR4 contributes to the implication of bone marrow in cancer metastasis. Future Oncol. 10 (5), 749–759. 10.2217/fon.13.193 24799056

[B82] Shin E. Koo J. S. (2020). The role of adipokines and bone marrow adipocytes in breast cancer bone metastasis. Int. J. Mol. Sci. 21 (14), 4967. 10.3390/ijms21144967 32674405 PMC7404398

[B83] Singh R. Stockard C. R. Grizzle W. E. Lillard J. W. Jr. Singh S. (2011). Expression and histopathological correlation of CCR9 and CCL25 in ovarian cancer. Int. J. Oncol. 39 (2), 373–381. 10.3892/ijo.2011.1059 21637913 PMC3760589

[B84] Slamon D. J. Neven P. Chia S. Jerusalem G. De Laurentiis M. Im S. (2021). Ribociclib plus fulvestrant for postmenopausal women with hormone receptor-positive, human epidermal growth factor receptor 2-negative advanced breast cancer in the phase III randomized MONALEESA-3 trial: updated overall survival. Ann. Oncol. 32 (8), 1015–1024. 10.1016/j.annonc.2021.05.353 34102253

[B85] Sung H. Ferlay J. Siegel R. L. Laversanne M. Soerjomataram I. Jemal A. (2021). Global cancer statistics 2020: GLOBOCAN estimates of incidence and mortality worldwide for 36 cancers in 185 countries. CA Cancer J. Clin. 71 (3), 209–249. 10.3322/caac.21660 33538338

[B86] Tanaka G. Nakase I. Fukuda Y. Masuda R. Oishi S. Shimura K. (2012). CXCR4 stimulates macropinocytosis: implications for cellular uptake of arginine-rich cell-penetrating peptides and HIV. Chem. Biol. 19 (11), 1437–1446. 10.1016/j.chembiol.2012.09.011 23177198

[B87] Tavassoli M. (2008). The marrow‐blood barrier. Br. J. Haematol. 41 (3), 297–302. 10.1111/j.1365-2141.1979.tb05862.x 371662

[B88] Teicher B. A. Fricker S. P. (2010). CXCL12 (SDF-1)/CXCR4 pathway in cancer. Clin. Cancer Res. 16 (11), 2927–2931. 10.1158/1078-0432.Ccr-09-2329 20484021

[B89] Ubukata H. Motohashi G. Tabuchi T. Nagata H. Konishi S. Tabuchi T. (2011). Overt bone metastasis and bone marrow micrometastasis of early gastric cancer. Surg. Today 41 (2), 169–174. 10.1007/s00595-010-4389-7 21264750

[B90] Umer M. Mohib Y. Atif M. Nazim M. (2018). Skeletal metastasis in renal cell carcinoma: a review. Ann. Med. Surg. (Lond) 27, 9–16. 10.1016/j.amsu.2018.01.002 29511536 PMC5832646

[B91] Wang D. Luo Y. Shen D. Yang L. Liu H. Y. Che Y. Q. (2019). Clinical features and treatment of patients with lung adenocarcinoma with bone marrow metastasis. Tumori 105 (5), 388–393. 10.1177/0300891619839864 30931812

[B92] Wang Y. C. Chang P. Y. Yao N. S. (2009). Bone marrow necrosis caused by metastatic colon cancer. J. Clin. Oncol. 27 (23), e48. 10.1200/JCO.2008.21.3140 19470920

[B93] Weilbaecher K. N. Guise T. A. McCauley L. K. (2011). Cancer to bone: a fatal attraction. Nat. Rev. Cancer 11 (6), 411–425. 10.1038/nrc3055 21593787 PMC3666847

[B94] Weiss A. R. Lyden E. R. Anderson J. R. Hawkins D. S. Spunt S. L. Walterhouse D. O. (2013). Histologic and clinical characteristics can guide staging evaluations for children and adolescents with rhabdomyosarcoma: a report from the Children's Oncology Group Soft Tissue Sarcoma Committee. J. Clin. Oncol. 31 (26), 3226–3232. 10.1200/JCO.2012.44.6476 23940218 PMC3757291

[B95] Wool G. D. Deucher A. (2015). Bone marrow necrosis: ten-year retrospective review of bone marrow biopsy specimens. Am. J. Clin. Pathol. 143 (2), 201–213. 10.1309/AJCP0TN1MCMOLMPK 25596246

[B96] Wu Y. Ni J. Chang X. Zhang X. Zhang L. (2020). Successful treatment of pyrotinib for bone marrow metastasis induced pancytopenia in a patient with non-small-cell lung cancer and ERBB2 mutation. Thorac. Cancer 11 (7), 2051–2055. 10.1111/1759-7714.13480 32458584 PMC7327666

[B97] Xiao L. Luxi S. Ying T. Yizhi L. Lingyun W. Quan P. (2009). Diagnosis of unknown nonhematological tumors by bone marrow biopsy: a retrospective analysis of 10,112 samples. J. Cancer Res. Clin. Oncol. 135 (5), 687–693. 10.1007/s00432-008-0503-2 18956213 PMC12160163

[B98] Xu L. Guo F. Song S. Zhang G. Liu Y. Xie X. (2014). Trastuzumab monotherapy for bone marrow metastasis of breast cancer: a case report. Oncol. Lett. 7 (6), 1951–1953. 10.3892/ol.2014.1999 24932266 PMC4049744

[B99] Yang R. Jia L. Lu G. Lv Z. Cui J. (2022a). Symptomatic bone marrow metastases in breast cancer: a retrospective cohort study. Front. Oncol. 12, 1042773. 10.3389/fonc.2022.1042773 36605432 PMC9808780

[B100] Yang R. Lu G. Lv Z. Jia L. Cui J. (2022b). Different treatment regimens in breast cancer visceral crisis: a retrospective cohort study. Front. Oncol. 12, 1048781. 10.3389/fonc.2022.1048781 36330468 PMC9623315

[B101] Yip R. K. H. Rimes J. S. Capaldo B. D. Vaillant F. Mouchemore K. A. Pal B. (2021). Mammary tumour cells remodel the bone marrow vascular microenvironment to support metastasis. Nat. Commun. 12 (1), 6920. 10.1038/s41467-021-26556-6 34836954 PMC8626461

[B102] Yoshioka K. Shimizu H. Yokoo S. Andachi H. (1992). Disseminated carcinomatosis of bone marrow from submucosal carcinoma in adenoma of the rectum. Intern Med. 31 (8), 1056–1059. 10.2169/internalmedicine.31.1056 1477466

[B103] Zhai X. Wang C. Li S. Cao T. Du G. Zhang Y. (2022). Bone marrow metastasis from advanced gastric cancer complicated with disseminated intravascular coagulation: a highly aggressive but manageable disease subtype. Cancer Commun. (Lond) 42 (4), 350–354. 10.1002/cac2.12277 35167192 PMC9017754

[B104] Zhang B. Zhang T. Jin L. Zhang Y. Wei Q. (2022). Treatment strategy of metastatic nasopharyngeal carcinoma with bone marrow involvement-A case report. Front. Oncol. 12, 877451. 10.3389/fonc.2022.877451 35747805 PMC9209652

[B105] Zych J. Polowiec Z. Wiatr E. Broniek A. Rowinska-Zakrzewska E. (1993). The prognostic significance of bone marrow metastases in small cell lung cancer patients. Lung Cancer 10 (3-4), 239–245. 10.1016/0169-5002(93)90184-y 8075969

